# Toward Physiological Detection of a “Just-Right” Challenge Level for Motor Learning in Immersive Virtual Reality: Protocol for a Cross-Sectional Study

**DOI:** 10.2196/55730

**Published:** 2024-09-23

**Authors:** Samory Houzangbe, Martin Lemay, Danielle E Levac

**Affiliations:** 1 School of Rehabilitation Faculty of Medicine University of Montreal Montréal, QC Canada; 2 Centre de Recherche du Centre Hospitalier Universitaire Sainte-Justine Montréal, QC Canada; 3 Department of Physical Activity Sciences Faculty of Sciences Université du Québec à Montréal Montréal, QC Canada

**Keywords:** virtual reality, pediatric rehabilitation, physiological data, engagement, just-right challenge

## Abstract

**Background:**

Motor learning, a primary goal of pediatric rehabilitation, is facilitated when tasks are presented at a “just-right” challenge level—at the edge of the child’s current abilities, yet attainable enough to motivate the child in persistent efforts for success. Immersive virtual reality (VR) may be ideally suited for “just-right” task challenges because it enables precise adjustments of task parameters in motivating environments. Rehabilitation-specific VR tasks often use dynamic difficulty algorithms based on task performance to personalize task difficulty. However, these approaches do not consider relevant cognitive processes that could also impact “just-right” challenges, such as attention and engagement. Objective physiological measurement of these cognitive processes using wearable sensors could support their integration within “just-right” challenge detection and prediction algorithms. As a first step, it is important to explore relationships between objectively and subjectively measured psychophysiological states at progressively challenging task difficulty levels.

**Objective:**

This study aims to (1) evaluate the performance of wearable sensors in a novel movement-based motor learning immersive VR task; (2) evaluate changes in physiological data at 3 task difficulty levels; and (3) explore the relationship between physiological data, task performance, and self-reported cognitive processes at each task difficulty level.

**Methods:**

This study uses the within-participant experimental design. Typically developing children and youth aged 8-16 years will be recruited to take part in a single 90-minute data collection session. Physiological sensors include electrodermal activity, heart rate, electroencephalography, and eye-tracking. After collecting physiological data at rest, participants will play a seated unimanual immersive VR task involving bouncing a virtual ball on a virtual racket. They will first play for 3 minutes at a predefined medium level of difficulty to determine their baseline ability level and then at a personalized choice of 3 progressive difficulty levels of 3 minutes each. Following each 3-minute session, participants will complete a short Likert-scale questionnaire evaluating engagement, attention, cognitive workload, physical effort, self-efficacy, and motivation. Data loss and data quality will be calculated for each sensor. Repeated-measures ANOVAs will evaluate changes in physiological response at each difficulty level. Correlation analyses will determine individual relationships between task performance, physiological data, and self-reported data at each difficulty level.

**Results:**

Research ethics board approval has been obtained, and data collection is underway. Data collection was conducted on December 12, 2023, and April 12, 2024, with a total of 15 typically developing children. Data analysis has been completed, and results are expected to be published in the fall of 2024.

**Conclusions:**

Wearable sensors may provide insights into the physiological effects of immersive VR task interaction at progressive difficulty levels in children and youth. Understanding the relationship between physiological and self-reported cognitive processes is a first step in better identifying and predicting “just-right” task challenges during immersive VR motor learning interventions.

**International Registered Report Identifier (IRRID):**

DERR1-10.2196/55730

## Introduction

### Overview

Virtual reality (VR) systems enable users to interact with virtual environments using body movements. Virtual environments can be categorized as immersive or nonimmersive [1]. Immersive VR is viewed in a head-mounted display (HMD) that provides a stereoscopic 3D viewing medium in which visual display changes in a natural way with head movements [2]. User movements are tracked by handheld or body-worn sensors to enable interaction with virtual objects. In contrast, nonimmersive VR is viewed on a 2D flat-screen display. VR incorporates evidence-based motor learning principles (such as multisensory feedback and abundant repetitions) and engaging immersive environments that may motivate children to adhere to repeated practice [3-5]. The use of nonimmersive VR has demonstrated effectiveness toward a variety of motor outcomes in pediatric rehabilitation [6]. With recent advances in immersive VR interaction abilities and the development of more lightweight and lower-cost HMDs, the use of immersive VR has become more attractive in pediatric rehabilitation, as complete visual immersion may enhance presence [7,8]. Several studies have established the feasibility of using immersive VR in pediatric rehabilitation, and there is preliminary evidence for the effectiveness of immersive VR interventions in promoting motor learning outcomes in children with disabilities [5]. While more information about the safety of long-term use in children younger than 12 years of age is required, recommendations for use with this age group include short periods of use punctuated by frequent breaks [9]. While the integration of immersive VR in clinical practice is in its early stages, a greater understanding of the potential unique advantages of immersive VR as a therapeutic intervention may support efforts at evidence-based integration.

One potential benefit of immersive VR is its potential to achieve a “just-right” task difficulty level during motor rehabilitation. A “just-right” task challenge is [3]:

structured so children are required to persist with and problem solve tasks in order to achieve success, however, they are not so difficult that the child loses interest, gives up, or fails the challenge

The “just-right” challenge is an important rehabilitation concept, evident across different disciplines, populations, and functional goals [10]. The concept of the “just-right” challenge is associated with the “challenge point framework” developed by Guadagnoli and Lee [11]. The authors theorize that an optimal degree of functional task difficulty for an individual with a specific level of skill will lead to optimal learning conditions, with evidence demonstrating the effectiveness of practice at this “challenge point” to improve motor learning [12]. Related to this framework are efforts to examine psychological factors that influence practice adherence, such as motivation [13]. Therapists working with children who require repeated, long-term rehabilitation interventions may struggle to keep them motivated to engage in the persistent efforts required to improve skills beyond current ability levels [14]. Novel and engaging virtual tasks and environments can help in that regard. In addition, VR applications custom-developed specifically for rehabilitation enable precise task difficulty selection, titrating task parameters to meet individual children’s ability levels more precisely than is possible in the real world. For these reasons, immersive VR may provide an ideal practice modality to target “just-right” challenges during motor skill learning. Currently, therapists use objective task performance indicators and subjective judgments of a child’s affective state when making difficult decisions targeting “just-right” challenges. This makes the presence of a therapist necessary to propose a “just-right” challenge. Some VR applications use automatic dynamic difficulty algorithms with performance-based decision rules to adjust task difficulty based on performance, in a similar way to the challenge point framework [15,16]. These algorithms are useful in situations where the therapist is not present to contribute clinical judgment, such as in telerehabilitation. However, the therapist brings additional important judgments outside of optimal task difficulty. Since the “just-right” challenge depends not only on performance (ie, how successful the learner is at accomplishing the task) but also on the learner’s motivation to persist in their efforts to succeed, it makes sense to consider how children’s cognitive processes can contribute to a “just-right” challenge. For example, a task difficulty level that is outside of a child’s abilities might be “just-right” for a child who is motivated to succeed and who enjoys being challenged, while a different child might be discouraged by failure and require a difficulty level closer to their abilities. Having an objective means of measuring proxies of these cognitive processes could supplement performance results in algorithms determining the “just-right” challenge in telerehabilitation. Psychophysiology is defined as the scientific study of the relationship between physiological and cognitive processes [17]. Measuring psychophysiological state in real time is possible with wearable sensors. Houzangbe et al [18] present a novel method for quantifying the “just-right” challenge in immersive VR based on psychophysiological data and performance variables. The authors outline the potential variables of interest and present hypothesized thresholds for the “just-right” challenge. Wearable sensors are an active research area in the pediatric population, mainly explored for measuring activity level and movement for physical rehabilitation [19]. However, multiple barriers still limit their usage [20,21], including difficulty with the placement of adult-sized sensors and movement artifacts during intense activities. Very little research has been done on the use of physiological sensor wearables (eg, heart rate [HR] and electroencephalography [EEG]) with pediatric populations. Existing studies are conducted in a static condition [22], or are missing details about the potential impact of movement artifacts on data integrity [23]. More evidence is required to understand potential data loss and data quality issues in wearable sensors when children undertake movement-based tasks [24]. The protocol described in this paper for this pilot study is focused on the feasibility of detecting variation in psychophysiological states in children during a new motor learning task in immersive VR. As such, it is centered around capturing objectively differing difficulty levels rather than identifying a “just-right” challenge. The study is the first step in a larger long-term research goal to train machine learning models to identify “just-right” challenges based on task performance and psychophysiological data. Although the interest of this study is in the “just-right” challenge during motor skill learning in children with motor impairments, typically developing children are included in this pilot study to capture a wider range of performance abilities in immersive VR.

### Objectives and Hypotheses

#### Objective 1

The objective is to evaluate the performance of (1) the electrodermal activity (EDA) and HR sensor and (2) the EEG 13-lead sensor in children undertaking a new motor learning task in immersive VR.

#### Hypothesis 1.1

Data loss will be less than 10%.

#### Hypothesis 1.2

A total of 70% of collected data will meet predetermined thresholds for data quality.

#### Objective 2

The aim is to evaluate changes in physiological data at 3 immersive VR task difficulty levels.

#### Hypothesis 2

EDA number of peaks per minute, HR, level of engagement, cognitive workload and concentration measured through EEG, average time of eye pursuit of the interactable objects, and average number of eye blinks per minute will differ between difficulty levels.

#### Objective 3

The aim is to explore the relationship between physiological data, task performance, and self-reported measures of engagement, cognitive workload, physical effort, and attention or focus at each task difficulty level.

#### Hypothesis 3.1

Self-report ratings will correlate positively with their corresponding physiological data at each of the 3 difficulty levels. Specifically, first, the EEG engagement index will positively correlate with the self-reported engagement score. Second, EEG cognitive workload will positively correlate with the self-reported cognitive workload score. Third, EEG concentration and average time of eye pursuit data will positively correlate with the self-reported concentration score. The average blink rate will negatively correlate with the self-reported concentration score. Finally, physical activity measured through acceleration data will positively correlate with self-reported physical effort scores.

#### Hypothesis 3.2

Levels of arousal, measured through HR and EDA data, will correlate negatively with task performance.

## Methods

### Ethical Considerations

This research project has been approved by the research ethics board of the Sainte-Justine University Hospital Research Center (2022-3881). We will send the informed consent form via email to potential participants; signing will occur at the study session after providing the opportunity to ask questions. The informed consent document explains why participants are invited to participate, the purpose of the project, the number of participants involved, and the age range. Finally, there is an optional section allowing participants to choose whether they agree to the secondary usage of their data for future research and whether their data will be made available.

### Privacy and Confidentiality Protection

Participant data will be deidentified using codes, and only the research team will have access to the code linking participants’ information to the collected data. On the informed consent form, parents have the option to grant permission for the use of deidentified images and videos of the participants for scientific communication and training purposes.

### Setting

Data collection will take place at the Technopôle for Pediatric Rehabilitation of the Marie Enfant Children’s Readaptation Center of the Sainte-Justine University Hospital Center in Montréal, Canada.

### Study Design

This study uses the repeated measure within-participant experimental design.

### Participants

#### Inclusion and Exclusion Criteria

A total of 15 typically developing children aged between 8 and 16 will be invited to participate. Inclusion criteria are cognitive, visual, and auditory abilities necessary to follow instructions and interact with the VR task. Exclusion criteria are photosensitive seizures, visuospatial deficiencies, and known cardiac problems. Interested participants will complete the cybersickness susceptibility questionnaire’s non–time-sensitive questions [25]. Since the experiment is performed seated, and the virtual environment does not move independently of the head movements, there is a limited risk of cybersickness. Children displaying 4 or more (out of 18 total) indicators of susceptibility to cybersickness (any “yes” answer in the yes-no questions and any rating of 1 in the Likert scales) will be excluded from participation.

#### Recruitment

Typically developing children will be recruited through social media advertisements. Participants and their parents will provide assent and consent, respectively.

#### Sample Size Calculation

This pilot study is not powered for effectiveness. For the analysis of repeated measure correlations, using the method developed by Bakdash and Marusich [26], with a presumed strong effect of task difficulty, 3 data points (the 3 different levels of difficulty), 15 participants in total are sufficient to reach a power of 0.80. This pilot study will determine the effect size to power a subsequent larger data collection.

### Novel Motor Learning Task in Immersive VR: Ball Bounce

**Figure 1:**
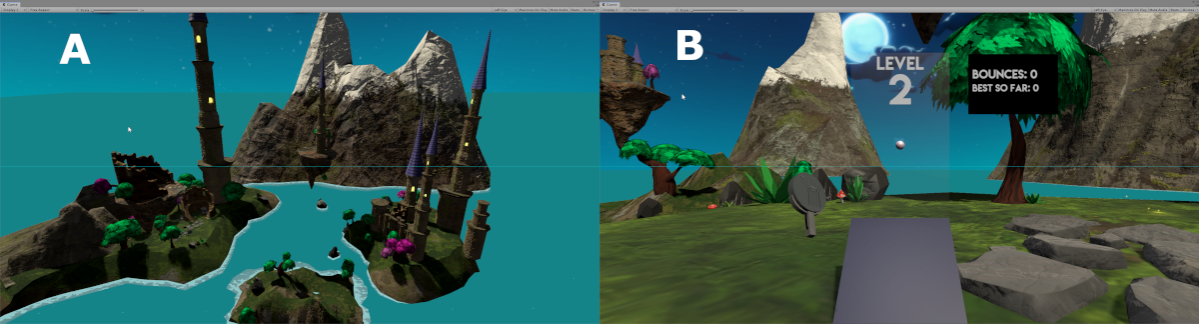
(A) The virtual environment. (B) The participant’s view.

Ball bounce is a custom-developed unimanual task built in Unity3D (Unity Technologies) that requires the player to consecutively bounce a virtual ball on a virtual paddle. The task takes place in a fantasy environment composed of floating islands and castles. The VR controller is represented in the virtual environment by a paddle (Figure 1). The user views a ping-pong–sized ball hovering directly in front of them at their eye level. When the user squeezes the controller trigger finger, the countdown starts and the ball is dropped. When the paddle enters in contact with the ball then the ball will bounce according to regular physics reaction. If the ball hits the ground, it disintegrates, and a new ball is generated and appears at eye level of the player at its initial position. A trial is started when a new ball is generated and starts falling. The trial ends when the ball hits the floor, automatically triggering a new ball to appear. Consecutive ball bounces during each trial are counted in the scoreboard and the highest number of bounces is displayed.

Task difficulty is modulated through Unity’s physics engine global gravity parameter. The ball is coded as a physical object and gravity modulates its speed. There are 10 difficulty levels, ranging from 10% of real-life gravity to 100% of real-life gravity; each progressive difficulty level has a 10% step increase. The virtual environment does not change across difficulty levels. The impact of gravity manipulation on perceived task difficulty was confirmed through preliminary testing with 5 typically developing children.

### Study Procedures

#### Overview

**Figure 2:**
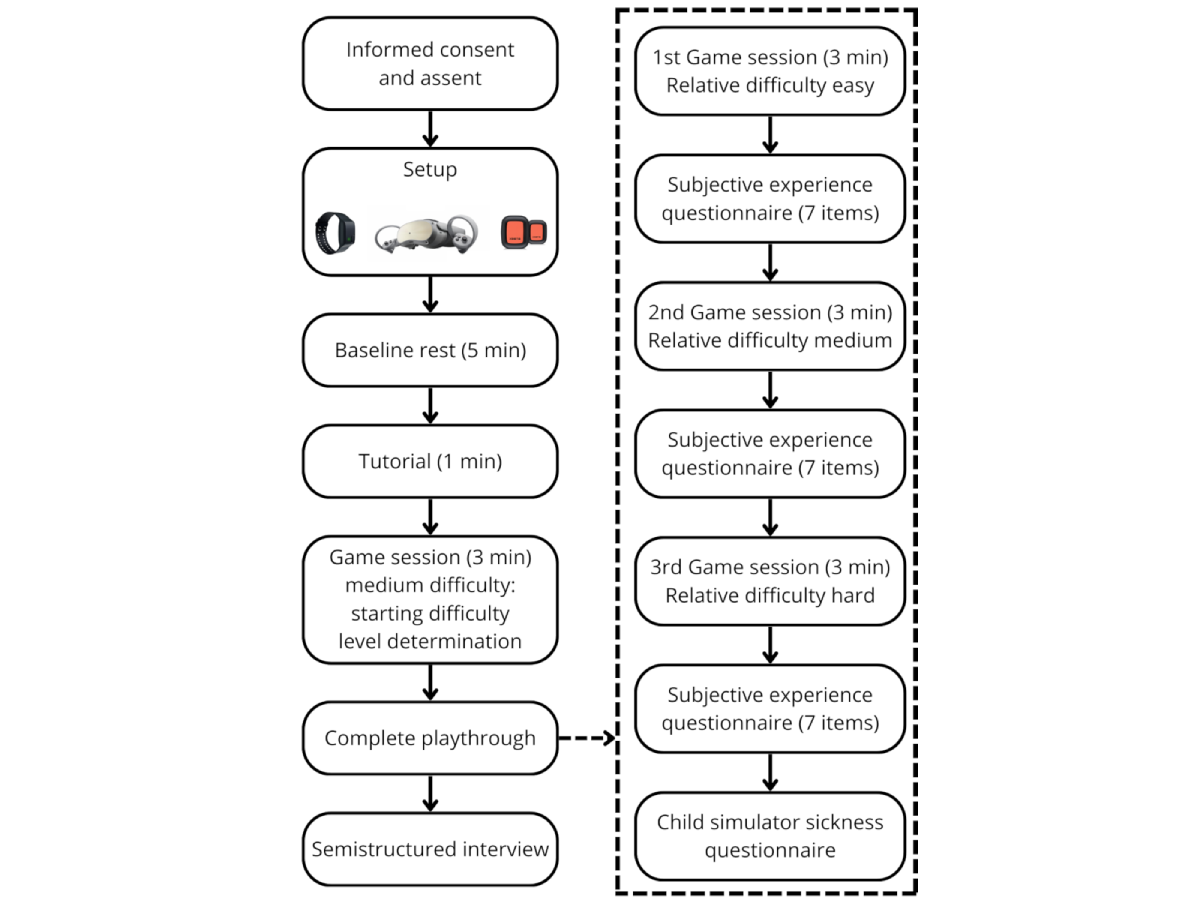
Overview of the data collection protocol.

Figure 2 outlines the study protocol. After completing a study demographic form, the participant is outfitted with the HMD and the data collection devices.

Participants will be asked to sit still for 5 minutes to calculate baseline resting state data. They will then receive task instructions Participants play with their dominant hand. All participants are asked to limit the movements of the nontask hand. Participants will first complete a 1-minute tutorial to familiarize them with the environment and the gameplay. The tutorial is set at a minimal level of difficulty (gravity is set to 10% of its real value). When participants are ready, they can click any button on the controller to start the game.

**Figure 3:**
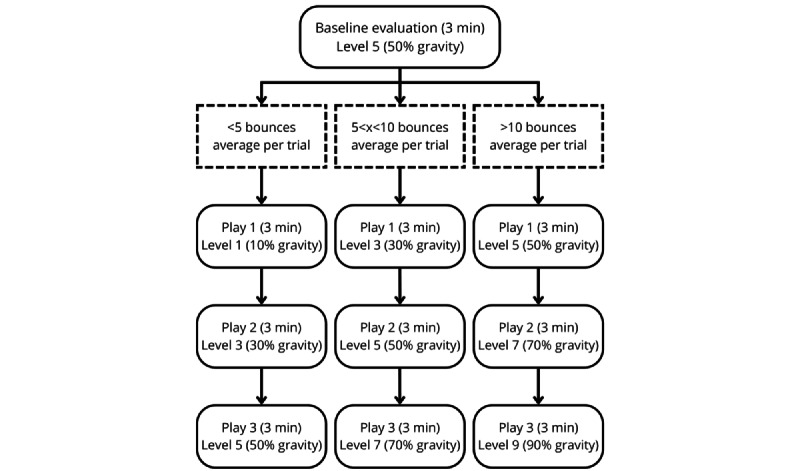
Difficulty selection and progression depending on baseline evaluation.

Participants will play for 3 minutes at the medium level of difficulty (gravity set to 50%) to evaluate their baseline task abilities. Depending on the participant’s performance during this baseline session, the starting difficulty will be set at a very easy level, an easy level, or a medium level. This personalization optimizes task difficulty progression to participant abilities. Visual representation of the difficulty selection and evolution is detailed in Figure 3. Participants then complete three 3-minute gameplay sessions at progressively challenging gravity manipulation levels, with breaks in between sessions. This number of gameplay sessions was chosen to limit the effects of fatigue. We chose not to counterbalance task difficulty presentation in order to better reflect real-world task learning conditions in which instructors increase task difficulty parameters as performance improves.

After each 3-minute play session, the participant removes the HMD and answers a study-specific questionnaire asking them to rate agreement with 7 statements (1 statement per construct) about their engagement, attention or focus, cognitive workload, physical effort, self-efficacy, and motivation. Statements are presented on a 7-item Likert scale with anchors on “strongly disagree” and “strongly agree.” First, engagement—2 questions inspired by the Independent Television Commission-Sense Of Presence Inventory [27] whose scores are averaged as (1) I enjoyed myself during this level and (2) I felt involved in the displayed environment. Second, attention or focus—I am completely focused on the task at hand (question derived from the Flow Short Scale [28]). Third, cognitive workload—during the task I did a lot of thinking and figuring out (questions derived from the revised National Aeronautics and Space Administration [NASA] Task Load Index [TLX] for children [29]). Fourth, physical effort—during the task I had to do a lot of physical effort (question derived from the revised NASA TLX for children [29]). Fifth, self-efficacy—I think I can do well at a more difficult level. Finally, motivation—I wanted to work harder to improve my performance or I tried hard to improve my performance (question derived from the Pediatric Motivation Scale [30])

The resting period between each 3-minute play session is between 3 and 5 minutes, and participants can request a longer break as needed. At the end of the session, participants complete the Child Simulator Sickness Questionnaire [31]. Participants will receive a CAD $25 (US $1=CAD $1.37) gift card as a token of appreciation for their participation in the study.

#### Data Collection Instruments

##### Task Performance

Participants will wear a Pico 4 Enterprise HMD. Performance data are collected within the Unity task—for each trial, the position of the paddle when the ball hits it and the maximum elevation of the ball after each hit is captured. The number of consecutive hits is calculated. To be considered as a valid bounce the ball must reach a height of at least 15 cm. Task performance is defined as the ratio of error over the total number of bounces attempted (number of errors divided by [number of bounces + number of errors]) during the 3-minute trial.

##### EEG Analysis

A Kaptics (Corporation) EEG with 12 Ag/AgCl dry electrodes is integrated into the Pico4 headgear. The electrodes are placed in the configuration—Fp1, Fp2, AFz (bias), F3, Fz, F4, C3, Cz (reference), C4, O1, Oz, O2. The Kaptics custom-developed application computes the psychophysiological metrics of (1) mental engagement level, measured with the engagement index, which is calculated through beta and alpha bands, based on the work of Coelli et al [32]; (2) cognitive workload, which is calculated through changes in theta and alpha bands, based on the work of Di Flumeri et al [33] and Zammouri et al [34]; and (3) concentration, which is measured through changes in beta bands, based on the work of Lim et al [35].

##### Eye-Tracking

The Pico Neo 4 Enterprise has an integrated Tobii eye tracker. Oculometry correlates with attention to a task [36]. The average time per trial of eye pursuit of the ball will be used as a metric of focused attention. Eye pursuit will be computed as the amount of time when performing the VR task, during which the direction of the eyes intersects with the areas of interest (volumes englobing respectively the ball and the paddle). A lower blink rate will be indicative of a higher focused attention [36].

##### Movement Quantity

The continuous position of the virtual paddle in the virtual environment is saved at a frame rate of 60 Hz. From the consecutive 3D positions recorded, the total amount of displacement will be computed to get the total amount of motion during the virtual task.

##### EDA and HR

Participants will wear an Empatica E4 sensor equipped with a photoplethysmography sensor and an EDA sensor. EDA, a recognized marker of arousal [37], will be computed as the average number of peaks per minute for each 3-minute play period using pyEDA, an open-source Python toolkit (Python Software Foundation) [38]. HR is also a proxy for arousal [37]. HR in beats per minute will be averaged over each gameplay. The changes in the level of arousal will be computed to determine the intensity of the physiological response. With an optimal level of engagement, arousal should be average [37]. If arousal is too high it can be interpreted as a sign of frustration rather than engagement.

##### Upper Extremity Movement Quality

As an exploratory measure, participants will wear XSens DOT inertial measurement units on the active forearm and on the chest to capture upper limb movement smoothness. Movement smoothness is measured through acceleration data. Less smooth movements are characterized by fluctuations in velocity, causing local maxima in the velocity profile. It is classified as an acceleration metric since the calculation of this metric is in the acceleration domain [39]. The lower the number of local maxima recorded the smoother the movement is. The average number of local maxima per minute will be computed as an exploratory measure to look for differences in movement at the different levels of difficulty.

##### Data Processing

Empatica data are stored in the smartwatch during the experiment and then downloaded to a computer for analysis. EDA data will be filtered using a low-pass Butterworth filter (frequency of 1 Hz and order of 6). EEG signals processed by Kaptics are filtered using a band-pass filter between 0.5 and 45 Hz and then zero-mean normalized. Kaptics uses the subspace reconstruction method to remove motion artifacts [40].

### Analyses

#### Overview

Quantitative statistical analyses will be performed using SPSS (IBM Corp) for ANOVA, correlations and graphical output and R (R Foundation for Statistical Computing) for the usage of the repeated measure correlation (rmcorr) package [26]. Confidence level will be set at 95%. Descriptive statistics will be presented as counts, means, and standard differences by level of difficulty. Demographic categorial data will be presented as means and standard differences by age, gender, and Gross Motor Function Classification System level.

#### Objective 1

The aim is to evaluate the performance of the physiological sensors in children undertaking a new motor learning task in immersive VR.

##### Identify Data Loss

The acceptable data loss threshold per measure will be set as 10% of the total data collection time for each of the 3 gameplay sessions. The expected output of data differs by sensor according to frequency. The amount of recorded data, for each 3-minute level, over expected output will be computed to measure the percentage of data loss.

1. EDA: Empatica frequency is 4 Hz
2. HR: Empatica HR value frequency is 1 Hz
3. Eye-tracking: Data will be collected at a fixed frequency (60 Hz). When the HMD is unable to accurately detect the eyes of the participants, the values are reported as 0.

##### Data Quality

For each 3-minute level, the amount of aberrant data will be computed for each sensor. The percentage of aberrant data over the total amount of collected data will then be computed.

First, the quality of EDA data is assessed following the two criteria of [41] (1) the total range of valid EDA data is 0.05-60 µS (data outside this range will be considered aberrant) and (2) an EDA change of more than ±10 µS/s between any 2 consecutive values is considered aberrant.

Second, HR—the minimal valid value of HR data is 60 beats per minute [42], while the estimated maximum valid value is 194 beats per minute [43]. Data outside this threshold will be considered aberrant. Changes in HR between any 2 consecutive values superior to ±3 beats per second will be considered aberrant [44].

#### Objective 2

Evaluate changes in physiological data at 3 immersive VR task difficulty levels. To examine intraindividual changes in averaged physiological data at baseline and the 3 difficulty levels, a repeated measure ANOVA will be performed for each dependent variable, with the level of difficulty as the within-subject factor. Sensitivity analysis will be done using the Friedman test, due to the small sample size. Eta squared will be reported as the measure of effect size.

#### Objective 3

The aim is to explore the relationship between physiological data, task performance, and self-reported measures of engagement, cognitive workload, physical effort, and attention or focus at each task difficulty level.

To determine the intraindividual relationship between physiological data and self-reported data, the Pearson product-moment correlation, and the Spearman rank correlation will be performed, to explore linear and monotonic relationships. The complete correlation matrix will be reported (with correlation coefficient as effect sizes and *P* values). At each difficulty level, correlations will be explored between (1) the level of engagement computed from the EEG data and the averaged self-reported score of engagement, (2) the level of attention computed from the EEG data and the self-reported score of attention, (3) the level of attention computed from the eye tracking data (time of pursuit and average blink rate) and the self-reported score of attention, (4) the level of cognitive workload computed from the EEG data and the self-reported score of cognitive workload, and (5) the average amount of movement of the virtual paddle and the self-reported score of physical effort.

Testing to evaluate parallel slopes between conditions will be undertaken [45]. If parallel slopes are identified, a repeated measure correlation (rmcorr) will be performed [26]. The rmcorr method can handle repeated measures data without violating independence assumptions or averaging data. It is ideally suited to assess association in intraindividual relationships between paired measures. Visual analysis [46], as well as statistical inference (regression coefficients and *P* values) will be reported. We will report visual analyses, as well as statistical inference (regression coefficients and *P* values) results.

## Results

Study recruitment is underway. The Centre hospitalier universitaire Sainte-Justine research ethics board approved this work (2022-3881) in the spring of 2023. Data collection was conducted on December 12, 2023, and April 12, 2024, with a total of 15 typically developing children. Data analysis has been completed, and results are expected to be published in the fall of 2024.

## Discussion

### Principal Findings

Targeting “just-right” task challenges during rehabilitation interventions is anchored in evidence-based motor learning principles [3]. Wearable sensors can enable objective measurement of psychophysiological states related to difficulty progression during motor skill learning. A better understanding of wearable sensor task performance during movement-based tasks in children is required [24]. The current state of knowledge on wearable sensor use in pediatric rehabilitation is limited to inertial measurement units and accelerometers [19]. Very few studies have explored the potential of wearable physiological sensors to understand children’s engagement during VR-based interactions [22]. This study will evaluate relationships between physiological data and children’s self-reports during the practice of a novel motor learning task in immersive VR at different task difficulty levels. Findings from this pilot study will inform subsequent work, which could include collecting physiological and self-report data with a child with a motor impairment who progresses at their own pace through task difficulty levels in immersive VR and asking children to self-identify when they perceive the challenge to be “just-right.” If objective and subjective data are correlated, this training data could be used to build a machine learning model to predict the “just-right” challenge based on the combination of thresholds of different variables, using the subjective self-report of the “just-right” challenge to correctly label the corresponding physiological data. As proposed by Houzangbe et al [18], combining the success rate of a task with psychophysiological levels of engagement, arousal, cognitive workload, and attention could lead to the objective identification of conditions necessary to reach personalized “just-right” challenges. In the longer term, embedding this model within immersive VR motor learning tasks may enable real-time decision-making about task difficulty level to achieve and maintain “just-right” task challenges. Subsequent work can also compare different types of VR tasks to assess the reproducibility of the results and their generalization.

### Potential Limitations

The small sample size of this pilot study limits the scope of the conclusions. Results will inform the calculation of the effect size required to power subsequent data collection. Using commercial physiological wearable sensors may lead to more compromised data quality as compared to medical-grade equipment. This limitation is balanced by cost and accessibility benefits. Choosing not to counterbalance task difficulty presentation introduces potential learning or fatigue effects. Using short house-made motivation and focus questionnaires is required as longer validated questionnaires are impractical following short gameplay sessions.

### Conclusions

This is the first step in a program of research exploring factors influencing children’s user experiences during motor skill learning in immersive VR. Immersive VR hardware and software are rapidly developing and lowering in cost, increasing their potential as an accessible telerehabilitation modality. If the difficulty of immersive VR tasks can be adapted to “just-right” challenges in the absence of therapeutic decision-making, they may be evidence-based, accessible, and personalized telerehabilitation interventions. Being able to identify, quantify, and predict “just-right” challenges can contribute to a future of precision rehabilitation [19] where VR, physiological sensors, and AI models can provide personalized interventions in clinic and home-based contexts.

## Abbreviations

EDA: electrodermal activity
EEG: electroencephalography
HMD: head-mounted display
HR: heart rate
NASA: National Aeronautics and Space Administration
TLX: Task Load Index
VR: virtual reality

## Data Availability

The datasets generated and analyzed during this study are available from the corresponding author upon reasonable request.

## References

[1] Fusco A, Tieri G (2022). Challenges and perspectives for clinical applications of immersive and non-immersive virtual reality. J Clin Med.

[2] Weiss PL, Keshner EA, Levin MF (2014). Virtual Reality for Physical and Motor Rehabilitation.

[3] Levac DE, Sveistrup H, Weiss PL, Keshner EA, Levin MF (2014). Motor learning and virtual reality. Virtual Reality for Physical and Motor Rehabilitation.

[4] Holt CJ, McKay CD, Truong LK, Le CY, Gross DP, Whittaker JL (2020). Sticking to it: a scoping review of adherence to exercise therapy interventions in children and adolescents with musculoskeletal conditions. J Orthop Sports Phys Ther.

[5] Demers M, Fung K, Subramanian SK, Lemay M, Robert MT (2021). Integration of motor learning principles into virtual reality interventions for individuals with cerebral palsy: systematic review. JMIR Serious Games.

[6] Vieira C, da Silva Pais-Vieira CF, Novais J, Perrotta A (2021). Serious game design and clinical improvement in physical rehabilitation: systematic review. JMIR Serious Games.

[7] Villani D, Riva F, Riva G (2007). New technologies for relaxation: the role of presence. Int J Stress Manag.

[8] Riva G, Mantovani F, Gaggioli A (2004). Presence and rehabilitation: toward second-generation virtual reality applications in neuropsychology. J Neuroeng Rehabil.

[9] Kaimara P, Oikonomou A, Deliyannis I (2022). Could virtual reality applications pose real risks to children and adolescents? A systematic review of ethical issues and concerns. Virtual Real.

[10] Poulsen AA, Rodger S, Ziviani JM (2006). Understanding children's motivation from a self‐determination theoretical perspective: implications for practice. Aus Occup Therapy J.

[11] Guadagnoli MA, Lee TD (2004). Challenge point: a framework for conceptualizing the effects of various practice conditions in motor learning. J Mot Behav.

[12] Ashouri S, Letafatkar A, Thomas AC, Yaali R, Kalantari M (2023). The challenge point framework to improve stepping reaction and balance in children with hemiplegic cerebral palsy: a case series study. J Pediatr Rehabil Med.

[13] Hodges NJ, Lohse KR (2022). An extended challenge-based framework for practice design in sports coaching. J Sports Sci.

[14] Chiarello LA, Palisano RJ, Avery L, Hanna S, On Track Study Team (2021). Longitudinal trajectories and reference percentiles for participation in family and recreational activities of children with cerebral palsy. Phys Occup Ther Pediatr.

[15] Valencia Y, Majin J, Guzmán D, Londoño J (2018). Dynamic difficulty adjustment in virtual reality applications for upper limb rehabilitation.

[16] Huber T, Mertes S, Rangelova S, Flutura S, André E (2021). Dynamic difficulty adjustment in virtual reality exergames through experience-driven procedural content generation.

[17] Browne T, Friedman HS (2016). Biofeedback and neurofeedback. Encyclopedia of Mental Health (Second Edition).

[18] Houzangbe S, Zejli Y, Lemay M, Levac D (2023). Quantifying individual and contextual factors that contribute to just-right challenge in an immersive virtual reality pediatric rehabilitation task: a protocol.

[19] Lang CE, Barth J, Holleran CL, Konrad JD, Bland MD (2020). Implementation of wearable sensing technology for movement: pushing forward into the routine physical rehabilitation care field. Sensors (Basel).

[20] Lobo MA, Hall ML, Greenspan B, Rohloff P, Prosser LA, Smith BA (2019). Wearables for pediatric rehabilitation: how to optimally design and use products to meet the needs of users. Phys Ther.

[21] Louie DR, Bird M, Menon C, Eng JJ (2020). Perspectives on the prospective development of stroke-specific lower extremity wearable monitoring technology: a qualitative focus group study with physical therapists and individuals with stroke. J Neuroeng Rehabil.

[22] Apicella A, Arpaia P, Giugliano S, Mastrati G, Moccaldi N (2021). High-wearable EEG-based transducer for engagement detection in pediatric rehabilitation. Brain Comput Interfaces.

[23] Vaughn J, Gollarahalli S, Shaw RJ, Docherty S, Yang Q, Malhotra C, Summers-Goeckerman E, Shah N (2020). Mobile health technology for pediatric symptom monitoring: a feasibility study. Nurs Res.

[24] Behere SP, Janson CM (2023). Smart wearables in pediatric heart health. J Pediatr.

[25] Freiwald JP, Göbel Y, Mostajeran F, Steinicke F (2020). The cybersickness susceptibility questionnaire: predicting virtual reality tolerance.

[26] Bakdash JZ, Marusich LR (2017). Repeated measures correlation. Front Psychol.

[27] Lessiter J, Freeman J, Keogh E, Davidoff J (2001). A cross-media presence questionnaire: the ITC-sense of presence inventory. Presence: Teleoperators Virtual Environ.

[28] Martin AJ, Jackson SA (2008). Brief approaches to assessing task absorption and enhanced subjective experience: examining ‘short’ and ‘core’ flow in diverse performance domains. Motiv Emot.

[29] Laurie-Rose C, Curtindale LM, Frey M (2017). Measuring sustained attention and perceived workload. Hum Factors.

[30] Tatla SK, Jarus T, Virji-Babul N, Holsti L (2015). The development of the Pediatric Motivation Scale for rehabilitation. Can J Occup Ther.

[31] Hoeft R, Vogel J, Bowers C (2016). Kids get sick too: a proposed child simulator sickness questionnaire. Proc Hum Factors Ergon Soc Annu Meet.

[32] Coelli S, Sclocco R, Barbieri R, Reni G, Zucca C, Bianchi A (2015). EEG-based index for engagement level monitoring during sustained attention. Annu Int Conf IEEE Eng Med Biol Soc.

[33] Di Flumeri G, Borghini G, Aricò Pietro, Sciaraffa N, Lanzi P, Pozzi S, Vignali V, Lantieri C, Bichicchi A, Simone A, Babiloni F (2018). EEG-based mental workload neurometric to evaluate the impact of different traffic and road conditions in real driving settings. Front Hum Neurosci.

[34] Zammouri A, Chraa-Mesbahi S, Moussa AA, Zerouali S, Sahnoun M, Tairi H, Mahraz AM (2017). Brain waves-based index for workload estimation and mental effort engagement recognition. J Phys Conf Ser.

[35] Lim S, Yeo M, Yoon G (2019). Comparison between concentration and immersion based on EEG analysis. Sensors (Basel).

[36] Adhanom IB, MacNeilage P, Folmer E (2023). Eye tracking in virtual reality: a broad review of applications and challenges. Virtual Real.

[37] Bian Y, Yang C, Gao F, Li H, Zhou S, Li H, Sun X, Meng X (2016). A framework for physiological indicators of flow in VR games: construction and preliminary evaluation. Pers Ubiquit Comput.

[38] Hossein Aqajari SA, Naeini EK, Mehrabadi MA, Labbaf S, Dutt N, Rahmani AM (2021). pyEDA: an open-source Python toolkit for pre-processing and feature extraction of electrodermal activity. Procedia Comput Sci.

[39] Scheltinga BL (2019). Suitable metrics for upper limb movement smoothness during stroke recovery. University of Twente Student Theses.

[40] Delorme A, Makeig S (2004). EEGLAB: an open source toolbox for analysis of single-trial EEG dynamics including independent component analysis. J Neurosci Methods.

[41] Kleckner IR, Jones RM, Wilder-Smith O, Wormwood JB, Akcakaya M, Quigley KS, Lord C, Goodwin MS (2018). Simple, transparent, and flexible automated quality assessment procedures for ambulatory electrodermal activity data. IEEE Trans Biomed Eng.

[42] What is a normal pulse rate?. British Heart Foundation.

[43] Verschuren O, Maltais DB, Takken T (2011). The 220-age equation does not predict maximum heart rate in children and adolescents. Dev Med Child Neurol.

[44] Enewoldsen NM (2016). Analysis of the quality of electrodermal activity and heart rate data recorded in daily life over a period of one week with an E4 wristband. University of Twente Student Theses.

[45] Tabachnick B, Fidell L (2018). Using Multivariate Statistics. 7 edition.

[46] Tukey J (1977). Exploratory Data Analysis. 1st edition.

